# Quantitative transport mapping of multi-delay arterial spin labeling MRI detects early blood perfusion alterations in Alzheimer’s disease

**DOI:** 10.1186/s13195-024-01524-6

**Published:** 2024-07-08

**Authors:** Yihao Guo, Liangdong Zhou, Yi Li, Gloria C. Chiang, Tao Liu, Huijuan Chen, Weiyuan Huang, Mony J. de Leon, Yi Wang, Feng Chen

**Affiliations:** 1 Department of Radiology, Hainan General Hospital (Hainan Affiliated Hospital of Hainan Medical University), No. 19, Xiuhua St, Xiuying Dic, Haikou, Hainan 570311 People’s Republic of China; 2 Department of Radiology, Brain Health Imaging Institute, Weill Cornell Medicine, 407 East 61 St ST, New York, NY 10066 USA; 3 Department of Radiology, Division of Neuroradiology, Weill Cornell Medicine, New York- Presbyterian Hospital, New York, NY USA; 4 Department of Neurology, Hainan General Hospital (Hainan Affiliated Hospital of Hainan Medical University), Haikou, China; 5 Department of Radiology, MRI Research Institute (MRIRI), Weill Cornell Medicine, New York, NY USA

**Keywords:** Cerebral blood flow (CBF), Quantitative transport mapping (QTM), Alzheimer’s disease, Early detection, Cognitive function, Perfusion imaging

## Abstract

**Background:**

Quantitative transport mapping (QTM) of blood velocity, based on the transport equation has been demonstrated higher accuracy and sensitivity of perfusion quantification than the traditional Kety’s method-based cerebral blood flow (CBF). This study aimed to investigate the associations between QTM velocity and cognitive function in Alzheimer’s disease (AD) using multiple post-labeling delay arterial spin labeling (ASL) MRI.

**Methods:**

A total of 128 subjects (21 normal controls (NC), 80 patients with mild cognitive impairment (MCI), and 27 AD) were recruited prospectively. All participants underwent MRI examination and neuropsychological evaluation. QTM velocity and traditional CBF maps were computed from multiple delay ASL. Regional quantitative perfusion measurements were performed and compared to study group differences. We tested the hypothesis that cognition declines with reduced cerebral blood perfusion with consideration of age and gender effects.

**Results:**

In cortical gray matter (GM) and the hippocampus, QTM velocity and CBF showed decreased values in the AD group compared to NC and MCI groups; QTM velocity, but not CBF, showed a significant difference between MCI and NC groups. QTM velocity and CBF showed values decreasing with age; QTM velocity, but not CBF, showed a significant gender difference between male and female. QTM velocity and CBF in the hippocampus were positively correlated with cognition, including global cognition, memory, executive function, and language function.

**Conclusion:**

This study demonstrated an increased sensitivity of QTM velocity as compared with the traditional Kety’s method-based CBF. Specifically, we observed only in QTM velocity, reduced perfusion velocity in GM and the hippocampus in MCI compared with NC. Both QTM velocity and CBF demonstrated a reduction in AD vs. controls. Decreased QTM velocity and CBF in the hippocampus were correlated with poor cognitive measures. These findings suggest QTM velocity as potential biomarker for early AD blood perfusion alterations and it could provide an avenue for early intervention of AD.

**Supplementary Information:**

The online version contains supplementary material available at 10.1186/s13195-024-01524-6.

## Introduction

Alzheimer’s disease (AD) is the leading cause of dementia amongst elderly adults, which typically manifests prominent symptoms of progressive decline in memory and multiple other cognitive domains [1]. The disease diminishes the quality of life for the patient, but also includes a heavy economic burden on society. Neurofibrillary tangles and amyloid-β neuritic plaques are the well-established pathological features of AD [2, 3]. However, AD pathogenesis is complex, involving multiple theories [4], and includes multiple risk factors [5]. Several studies have pointed out that vascular risk factors play an important role in the process of developing AD pathology [6, 7]. Previous studies have found that these vascular risk factors lead to vascular injury, resulting in cerebral perfusion alterations [8]. Moreover, it has been shown that neuronal damage leads to reduced demand for oxygen and glucose, thus secondarily reducing cerebral blood flow (CBF) [9, 10]. 

Perfusion quantification using magnetic resonance imaging (MRI) is based on modeling a tracer transport through tissue captured in time-resolved imaging, such as dynamic contrast enhanced (DCE), dynamic susceptibility contrast (DSC), and arterial spin labeling (ASL). DCE and DSC methods use the injected gadolinium-based contrast agents (GBCA) as tracers. The ASL method, alternatively, uses radiofrequency labeled water as an endogenous tracer and is widely applied for perfusion quantification in research and gaining increasing attention in clinical practice due to its wide availability and non-invasive manner [11]. Conventionally, these approaches for CBF quantification use Kety’s equation by relating the temporal change in tracer concentration to an arterial input function (AIF) for each voxel [12]. Since AIF at each voxel is not practically measurable, a single global AIF is assumed for blood perfusion to all brain regions and is known to have errors and violate the local mass conservation principle [13]. This commonly known AIF problem of conventional perfusion modeling has gained attention and encouraged the development of new approaches using spatiotemporal information for perfusion quantification [14]. To address this problem, we proposed to model changes in spatiotemporal tracer concentration according to the mass transport equation that utilizes spatial and temporal derivatives of the concentration without the selection of an AIF [13]. Blood flow velocity can be calculated fully automatedly by fitting four-dimensional (4D) dynamic tracer imaging data to the transport equation, which is termed as quantitative transport mapping (QTM) [13]. It has been demonstrated that (1) QTM velocity is more accurate than traditional CBF for blood perfusion quantification in silico validation; [13, 15] (2) QTM velocity has a significant value in identifying breast cancer malignancy, [16] nasopharyngeal cancer gene expressions [17], lung shunt fraction [18], and progressive liver disease stages [15]. 

Given its promising diagnostic value in various diseases, we applied this technique to AD in this work for the first time to evaluate its ability for early detection. We tested two major hypotheses: (1) QTM velocity is superior to CBF in the separation of clinical AD spectrum groups; (2) QTM model as compared with Kety’s method, offers better regional correlation with clinical cognitive performance measures.

## Materials and methods

### Subjects

This study was approved by the Ethics Committee of Hainan General Hospital in accordance with the Declaration of Helsinki. All participants and/or their respective Legally Authorized Representative (when applicable) provided their written informed consent.

A total of 176 subjects aged 55 to 90 years old were recruited from the community. All participants underwent neuropsychological tests and MRI examinations at Hainan General Hospital, Haikou, China. Exclusions included 43 participants who were unable to complete neuropsychological tests and 5 participants who could not remain still in the MRI or had severe artifacts in images leaving 128 eligible subjects. A diagnosis of probable AD was made based on the criteria set by the National Institute on Aging and Alzheimer’s Association (NIA-AA) [19] while a diagnosis of Mild Cognitive Impairment (MCI) was made according to Petersen [20]. The definition of cognitively healthy control in addition to clinical interview was corroborated by Mini-Mental State Examination (MMSE) score > 27 and a Clinical Dementia Rating (CDR) score of 0 [21]. 

### Neuropsychological tests and cognitive outcomes

To assess cognitive status, 4 neuropsychological tests were administered [22]. 

The MMSE is a 30-item screening tool used to summarize cognitive abilities including orientation, memory, attention, and language [23]. We utilized the total score in our analysis.

The Trail Making Test (TMT) A and B require participants to draw a line connecting circles that contain numbers (A) or letters and numbers (B) in ascending order [24]. The time needed to complete each test are indicators of processing speed and executive function.

In the Rey Auditory Verbal Learning Test (RAVLT), a list of 15 words is read 5 times. The participant is asked to recall the words after each presentation (immediate recall and learning). After a 20-minute delay, the participant is asked to recall the words again (delayed recall). We utilized the mean number of words recalled for the first 3 trials (immediate recall scores) as indicators of episodic memory and analyzed the total number of words recalled after the 20-minute delay (delayed recall score) [25]. 

In the semantic verbal fluency test (VFT), the participant is asked to name as many animals as possible in 60 s. We utilized the total number of animals named as an indicator of semantic fluency [26]. 

### MRI data acquisition

All participants underwent MR examinations using a 3.0T MR scanner (Prisma, Siemens) with a 64-channel head/neck receiver coil. The imaging protocol included a three-dimensional (3D) magnetization-prepared rapid acquisition gradient-echo (MPRAGE T1W) sequence for anatomical imaging and a 3D pseudo-continuous ASL sequence with multiple post label delay (mPLD) for perfusion quantification [27, 28]. Scanning parameters were as follows: (1) MPRAGE: echo time (TE) = 2.26 ms; repetition time (TR) = 2300 ms; Inversion time = 900 ms; flip angle = 8^o^; slice thickness = 1 mm; field of view (FOV) = 256 × 256 mm^2^; voxel size = 1 × 1 × 1 mm^3^; (2) pseudo-continuous ASL (PCASL): TE = 37.78 ms; TR = 4200 ms; five PLDs = 500, 1000, 1500, 2000, 2500 ms; slice thickness = 3 mm; FOV = 240 × 240 mm^2^; voxel size = 2.5 × 2.5 × 3 mm^3^; Routine MR sequences (T2W and T2-FLAIR) were also included to detect brain abnormalities.

## MRI data processing

### T1w based brain ROI parcellation

T1w MRI was regionally segmented using FreeSurfer (FS) version 7.1 [29] recon-all command for region of interest (ROI) parcellation. Individual ROIs defined by FS look-up-table (LUT) were combined bilaterally for the extraction of ROI values in CBF and QTM velocity maps. The CBF and QTM velocity were coregistered into FS T1w space before ROI value extraction. ROIs evaluated in this study include global cerebral cortex (GM), cerebral white matter (WM), deep gray matter (dGM), four cortical lobes (temporal (TL), frontal (FL), parietal (PL), occipital (OL)), and hippocampus (Hippo), which were shown in Supplementary material (Figure S1 and Tabel S1). To reduce the potential partial volume effect (PVE), all the ROIs used in this work were eroded 1 mm in FS space. We also have warped the CBF and QTM velocity into the Montreal Neurological Institute (MNI) space for the evaluation of group mean using the volume-based Advanced Normalization Tools (ANTs) package. [30]

### CBF mapping from multidelay ASL

CBF (ml/100 g/min) maps were reconstructed from the mPLD PCASL data using the *oxford_asl* command in BASIL tools included in FSL [31]. Specifically, the mPLD ASL data was first realigned using mcflirt in FSL with the M0 proton image as a reference [32]. The realigned image was distortion corrected using the anterior-posterior and posterior-anterior encoding reference images with top-up correction implemented in FSL to reduce the effect of air-tissue boundary distortion in EPI-based sequence [33]. The preprocessed mPLD ASL data was processed by subtracting the labeling image from the control image and then used the *oxford_asl* command for blood perfusion quantification with bolus time 1.5 s, PLDs = 0.5, 1, 1.5, 2, 2.5 s, T1 blood = 1.65 s, labeling efficiency = 0.85 and spatial smoothing regularization [34, 35]. The details of the kinetic model for CBF quantification using multi-delay ASL data are provided in the Supplementary material.

### QTM from multidelay ASL

The quantitative transport mapping of blood velocity was modeled by the mass conservation equation of the tracer [13, 15, 16]:

$$\eqalign{{\partial _t}c\left( {{\bf{\it{\bf r}}},t} \right) & = - \nabla \cdot \left( {c\left( {{\bf r},t} \right){\bf{\it{\bf u}}}\left( {\bf{\it{\bf r}}} \right)} \right) + \cr & \nabla \cdot \left( {D\left( {\bf{\it{\bf r}}} \right)\nabla c\left( {{\bf{\it{\bf r}}},t} \right)} \right) - \lambda c({\bf{\it{\bf r}}},t), \cr}$$

where $c\left(\varvec{r},t\right)$ is the tracer concentration at location $\varvec{r}$ and time $t$, $\varvec{u}\left(\varvec{r}\right)$ is the time-invariant tracer velocity, $D\left(\varvec{r}\right)$ is the apparent diffusion coefficient, and $\lambda$ is the signal decaying rate. For MR labelled endogenous water molecular in ASL data, the $\lambda =1/T1b$, and $T1b=1.65$ seconds is the T1 time of blood. For perfusion estimation, $D\left(\varvec{r}\right)$ could be considered negligible since diffusion effects are at a much slower rate than blood perfusion. The reconstruction of perfusion velocity is then performed following the optimization below [13, 15, 16]:

$${\bf{\it{\bf u}}} = argmi{n_{\bf{\it{\bf u}}}}\mathop {\sum \,\parallel }\limits_{t = 1}^{{N_t} - 1} \,{\partial _t}c + \nabla \cdot \left( {c{\bf{\it{\bf u}}}} \right) + \lambda c\parallel \,_2^2 + \alpha \parallel \nabla {\bf{\it{\bf u}}}\parallel {_1},$$

where $\alpha$ is the regularization parameter in the optimization to enforce a region-wise smooth solution. The optimization of the above minimization problem used an alternating direction method of multipliers (ADMM) with the conjugate gradient algorithm as a subroutine for solving the linear sub-problems and the regularization parameter $\alpha =0.05$ was determined by using the L-curve approach. [36, 37] The reconstruction processing of $\varvec{u}$ was performed using in-house code executed in MATLAB with realigned and top-upped multidelay ASL data. According to the previous work on QTM, the velocity vector $\varvec{u}$ is mean tracer velocity in capillaries across voxel surfaces with a typical unit as mm/sec in image space. [13] The magnitude of $\varvec{u}$ was denoted as $\parallel{\bf u}\parallel$ in L2 norm to represent the blood flow velocity in QTM.

### Statistical analysis

The statistical analyses were conducted using SPSS 20.0 and R Ver 4.3.2 in RStudio 2022. All significance tests were 2-sided with α = 0.05 as the significance threshold. Normality was assessed using the Shapiro-Wilk test for normality prior to testing group differences [38]. Continuous variables were expressed as means ± standard deviations. Analysis of variance and χ^2^ tests were used to investigate group differences in demographic and cognitive variables. CBF and QTM velocity values of investigated ROIs were compared among diagnostic groups using one-way analysis of covariance with age, sex, and years of education as covariates. Post-hoc multiple comparisons were performed to evaluate statistical differences between diagnostic groups. Correlation analyses were performed to investigate the relationships between regional CBF and QTM velocity across diagnoses. Finally, we assessed the effects of age and sex on blood perfusion and velocity using linear regression, and the association between cognitive score and perfusion measures using Pearson or Spearman’s correlation analysis. Note that all r reported are the correlation coefficient, and the p values reported are FDR adjusted for multiple comparisons [39]. 

## Results

### Clinical and demographic characteristics

Among 128 eligible subjects, there were 27 probable AD patients, 80 MCI patients, and 21 NC. According to the cutoff value of plasma Aβ42/Aβ40 [40], more than 77% MCI patients were presumed underlying AD with Aβ positive status. There was no significant difference in sex among the three groups (*p* = 0.213). Mean age was higher in the AD than the NC (*p* = 0.021) and MCI groups (*p* = 0.021), and years of education were higher in the NC than the MCI (*p* = 0.003) and AD groups (*p* = 0.002). Cognition scores including MMSE, immediate recall score, delayed recall score, TMT-A, TMT-B, and semantic fluency were significantly different among the three groups, consistent with the diagnoses (all *p* < 0.001). These results are summarized in Table 1.

**Table 1:** Demographics and clinical characteristics of the Study Population

	NC (*n* = 21)	MCI (*n* = 80)	Probable AD (*n* = 27)	F/χ2 value	*P* value
Age	67.95 ± 6.68	67.31 ± 6.15	72.44 ± 8.61	5.799	0.004
Gender %F	62	55	74	3.096	0.213
Education level (years)	13.81 ± 4.13	10.23 ± 3.79	9.48 ± 4.07	8.548	< 0.001
MMSE	29.05 ± 1.28	25.11 ± 3.90	14.46 ± 6.66	80.253	< 0.001
Immediate recall score	6.52 ± 1.76	4.44 ± 1.66	2.50 ± 1.10	36.076	< 0.001
Delayed recall score	6.38 ± 2.04	3.44 ± 2.47	1.23 ± 1.77	27.320	< 0.001
TMT-A (s)	62.43 ± 18.60	92.16 ± 43.50	210.50 ± 137.51	31.300	< 0.001
TMT-B (s)	146.10 ± 37.30	199.72 ± 68.46	495.92 ± 271.95	46.491	< 0.001
Semantic fluency	19.81 ± 4.20	14.00 ± 5.29	8.04 ± 3.68	33.855	< 0.001

### Group mean of QTM velocity and CBF

**Fig. 1:**
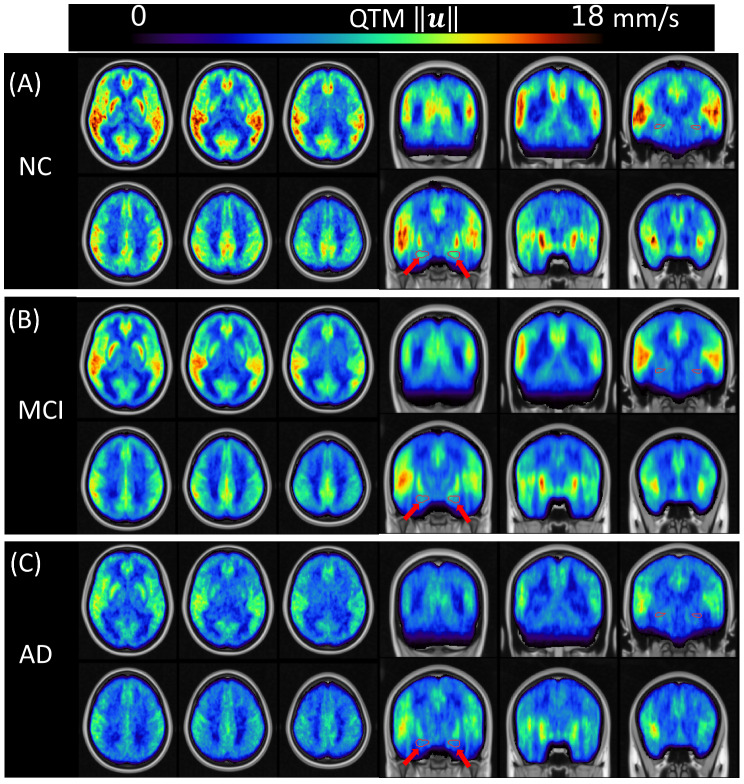
Group averaged QTM velocity in MNI template. (**A**), (**B**), and (**C**) are QTM velocity in NC, MCI, and AD, respectively. QTM velocity shows decreased pattern across NC, MCI, and AD groups in axial and coronal views. Red arrows point to the hippocampus in coronal view to show the QTM velocity across groups. We see a drastic QTM velocity reduction in the hippocampus from NC to MCI and AD

**Fig. 2:**
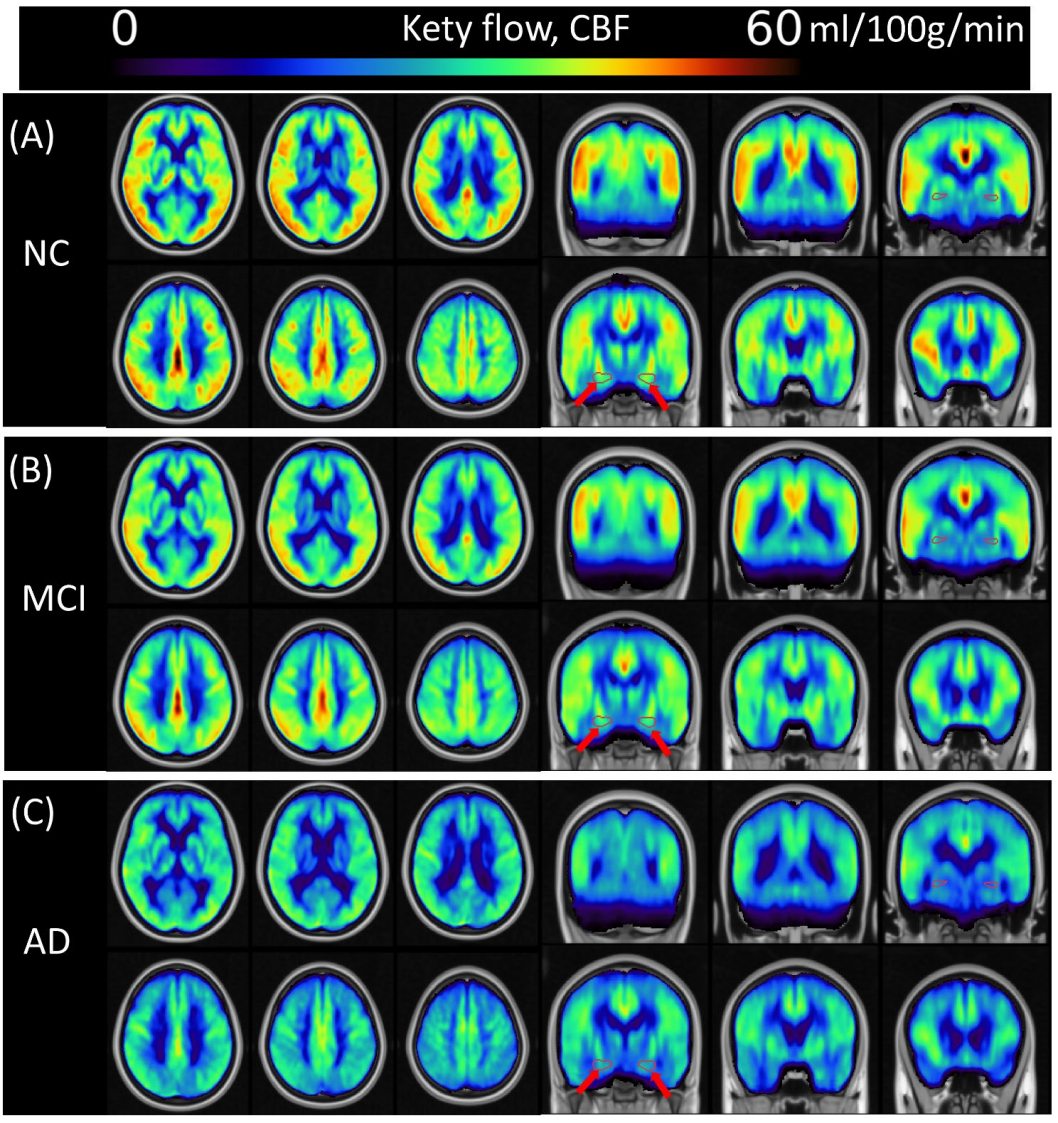
Group averaged CBF in MNI template. (**A**), (**B**), and (**C**) are CBF in NC, MCI, and AD, respectively. CBF shows decreased pattern across NC, MCI, and AD groups in axial and coronal views. Red arrows point to the hippocampus in coronal view to show the CBF across groups

Figure 1 presents the averaged maps of QTM velocity and Fig. 2 shows the CBF for NC, MCI, and AD groups in the MNI space. The whole-brain patterns of CBF and QTM velocity across the diagnostic groups were visually different in AD from those in NC and MCI. Both QTM velocity and CBF values across groups follow the order: NC > MCI > AD. The hippocampus region was highlighted by red arrows for perfusion comparison across groups.

### Group difference of regional QTM velocity and CBF

**Fig. 3:**
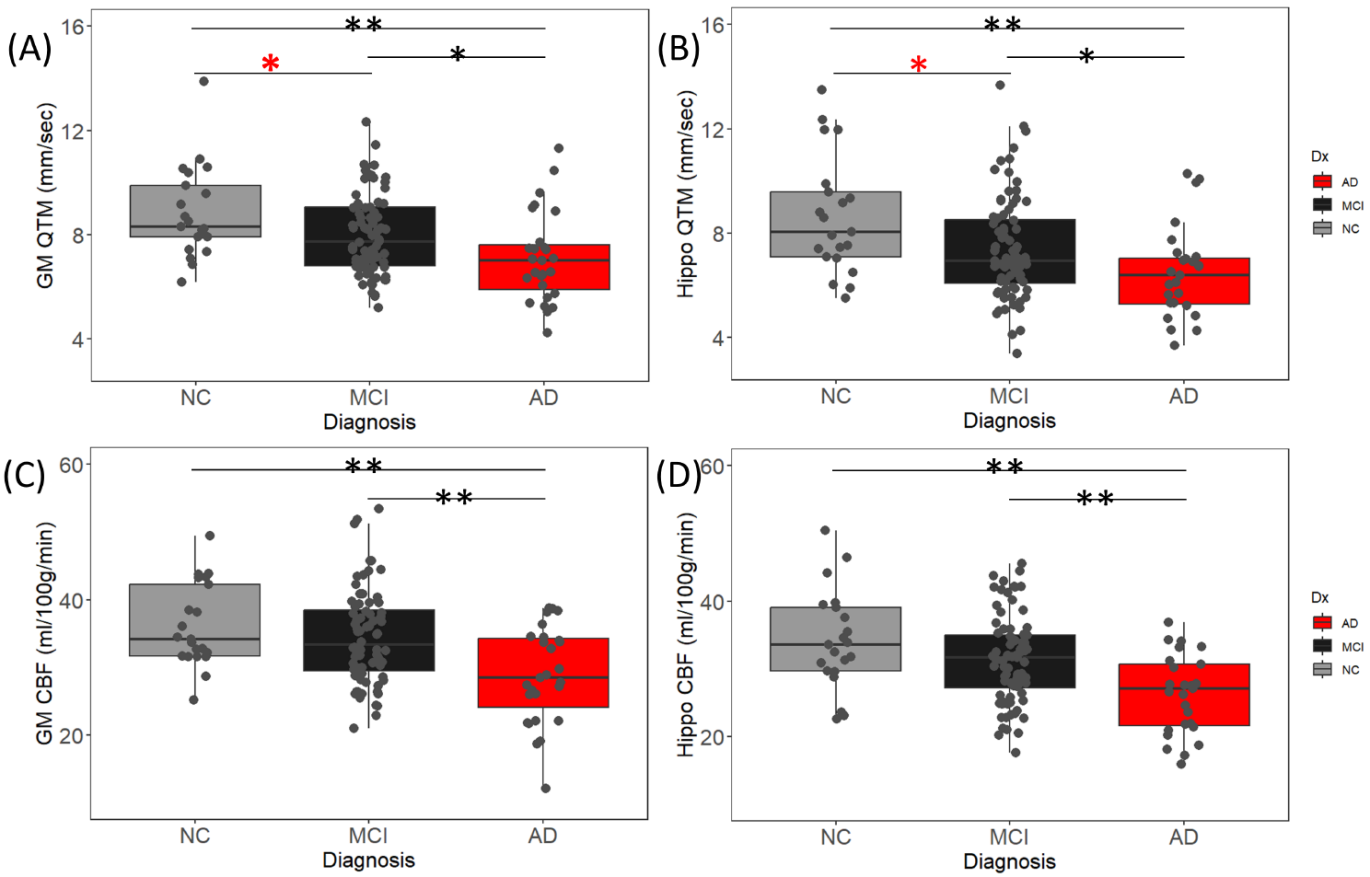
Pairwise group differences of regional QTM velocity and CBF. (**A**) and (**B**) are QTM velocity in GM and Hippo, respectively. (**C**) and (**D)** are CBF in GM and Hippo. QTM velocity shows group differences between NC, MCI, and AD in both regions. CBF only differs between AD and MCI or NC, but not between MCI and NC. Asterisks indicate significance: * indicates *p* < 0.05, ** indicates *p* < 0.01. The red asterisks highlight the significant QTM velocity reduction from NC to MCI, which was not applied to CBF

Figure 3 shows decreased QTM velocity values in MCI patients compared to NC in both GM (Fig. 3(A), NC: 8.834 ± 1.758 mm/s, MCI: 8.006 ± 1.539 mm/s, *p* = 0.039) and Hippo (Fig. 3(B), NC: 8.640 ± 2.253 mm/s, MCI: 7.360 ± 1.949 mm/s, *p* = 0.018). However, the CBF values were unable to distinguish MCI patients from NC (GM: *p* = 0.283, Hippo: *p* = 0.082). The results also revealed decreased QTM velocity value in AD patients compared to MCI and NC in GM (AD vs. MCI: *p* = 0.027; AD vs. NC: *p* = 0.001) and Hippo (AD vs. MCI: *p* = 0.035; AD vs. NC: *p* < 0.001), and decreased CBF value in GM (Fig. 3(C), AD vs. MCI: *p* < 0.001; AD vs. NC: *p* < 0.001) and Hippo (Fig. 3(D), AD vs. MCI: *p* < 0.001; AD vs. NC: *p* < 0.001). A summary of ROI values (mean ± standard deviation) for QTM velocity and CBF is presented in Table 2, and the p-value from one-way ANCOVA is listed in the last column of the table. Note that we also provided subject level QTM velocity and CBF map for their comparison in the Supplementary material (Figure S2).

**Table 2:** Comparisons of QTM and CBF values among three diagnostic groups in defined ROIs

	NC	MCI	Probable AD	F value	*P*
**QTM value (mm/sec)**					
GM	8.83 ± 1.76	8.01 ± 1.54	7.10 ± 1.72	5.325	**0.006**
WM	6.30 ± 1.45	5.76 ± 1.20	5.63 ± 1.23	1.803	0.169
dGM	9.46 ± 1.80	8.76 ± 1.77	7.87 ± 1.75	3.902	**0.023**
TL	8.54 ± 1.66	7.73 ± 1.46	6.79 ± 1.66	6.109	**0.003**
FL	8.55 ± 1.75	7.85 ± 1.46	7.09 ± 1.64	3.701	**0.028**
PL	9.65 ± 2.09	8.69 ± 2.00	7.43 ± 2.17	6.114	**0.003**
OL	7.41 ± 1.88	6.37 ± 1.58	6.11 ± 1.48	4.190	**0.017**
Hippo	8.64 ± 2.25	7.36 ± 1.95	6.43 ± 1.74	5.627	**0.005**
**CBF value (ml/100 g/min)**					
GM	36.15 ± 6.15	34.37 ± 6.79	28.88 ± 7.02	5.804	**0.004**
WM	23.02 ± 4.51	21.75 ± 4.90	19.70 ± 5.12	2.883	0.060
dGM	34.11 ± 6.50	31.72 ± 6.68	28.41 ± 7.16	4.452	**0.014**
TL	33.90 ± 6.29	32.66 ± 6.21	27.56 ± 6.82	5.487	**0.005**
FL	35.45 ± 6.52	33.11 ± 6.97	28.11 ± 7.43	5.038	**0.008**
PL	39.85 ± 6.64	38.13 ± 8.13	30.53 ± 8.15	7.535	**0.001**
OL	35.21 ± 8.07	34.07 ± 7.95	30.11 ± 10.35	2.155	0.120
Hippo	34.21 ± 7.36	31.44 ± 6.42	26.26 ± 5.79	7.063	**0.001**

### Age and sex effects of QTM velocity and CBF

**Fig. 4:**
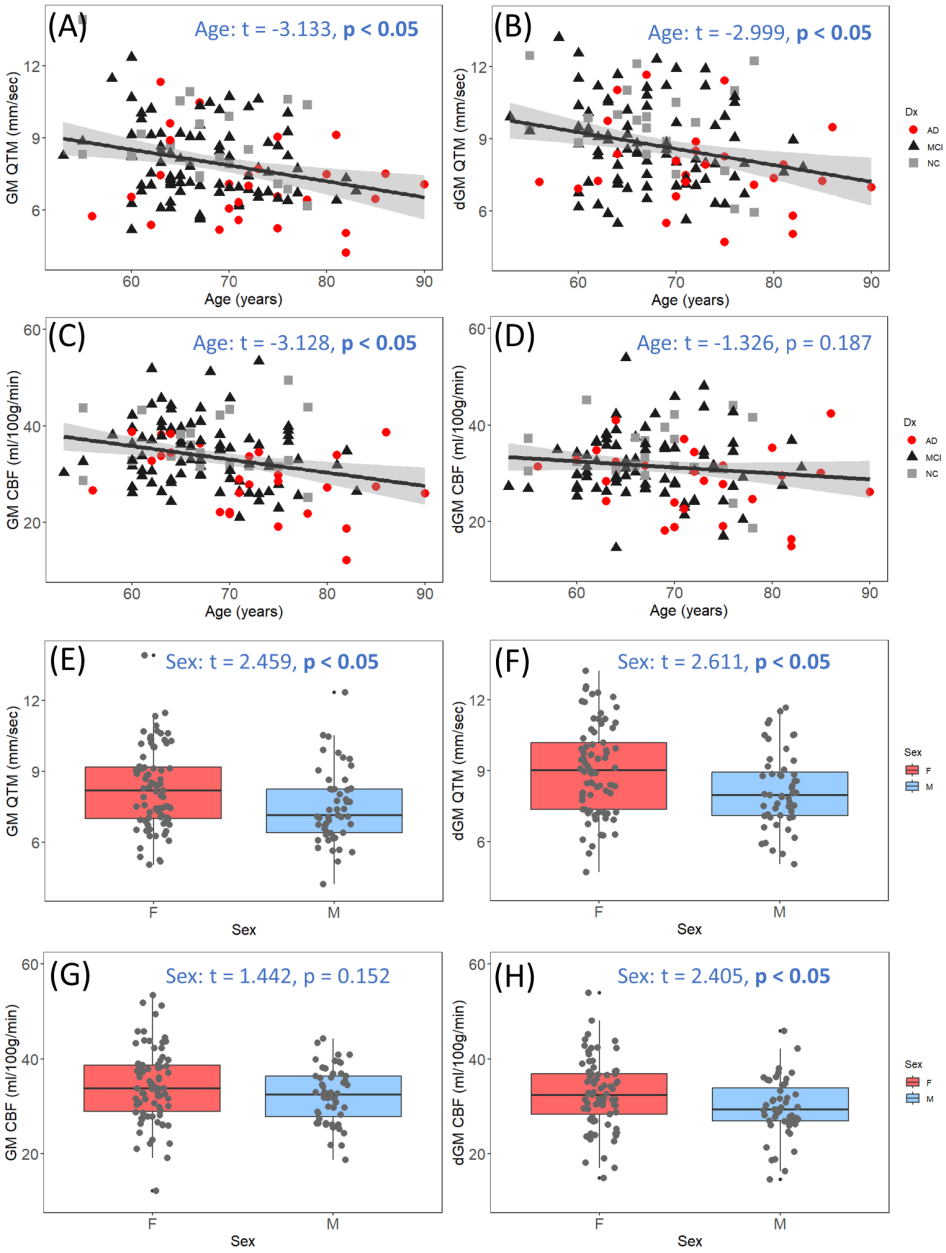
The age and sex effects of QTM velocity and CBF in cortical GM (GM) and deep GM (dGM). (**A**) to (**D**) are GM QTM velocity, dGM QTM velocity, GM CBF, and dGM CBF with age, respectively; (**E**) to (**H**) are GM QTM, dGM QTM velocity, GM CBF, and dGM CBF by sex. QTM velocity shows significant age and sex effects in both GM and dGM, while CBF only shows age effect in GM and sex effect in dGM. Statistics are from multiple linear regressions including both sex and age as independent variables in the model

The effects of age and sex on QTM velocity and CBF are presented in Fig. 4 in GM and dGM. Linear regression shows that QTM velocity in both GM and dGM significantly reduced with age (Fig. 4A and B) and male sex (Fig. 4E and F), with females having higher QTM velocity values than males (GM: age t = -3.133, *p* = 0.002, sex t = 2.459, *p* = 0.015; dGM: age t = -2.999, *p* = 0.003, sex t = 2.611, *p* = 0.010). In contrast, CBF in GM only shows a significant age effect, i.e., GM CBF decreases with age (Fig. 4C, t = -3.128, *p* = 0.002) but no sex effect (Fig. 4G, t = 1.442, *p* = 0.152), and the effect of sex is only significant in the dGM with higher CBF in females (Fig. 4H, t = 2.405, *p* = 0.018) but no age effect (Fig. 4D, t = -1.326, *p* = 0.187).

### Regional CBF and QTM velocity correlations

**Fig. 5:**
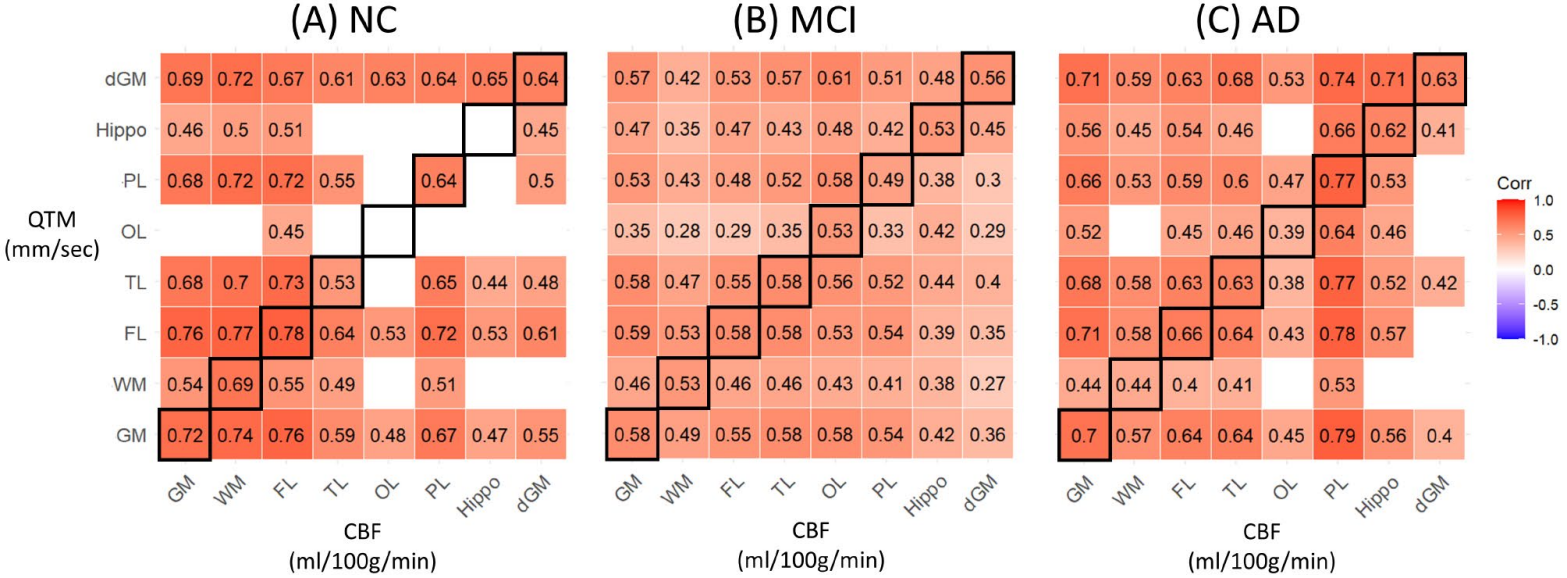
Correlation maps between QTM velocity (mm/sec) and CBF (ml/100 g/min) in multiple regions, including GM, WM, FL, TL, OL, PL, Hippo, and DeepGM. The numbers shown in the maps are the correlation coefficients. The correlation is lower in the MCI and AD groups compared to NC group. (**A**) correlation in NC group; (**B**) MCI group; (**C**) AD group. Note that the same ROI correlations between QTM velocity and CBF are highlighted by black boxes, and insignificant correlations are blanked

To investigate any inconsistencies between CBF and QTM velocity, we performed correlation analyses between regional CBF and QTM velocity across diagnostic groups. Results are shown in Fig. 5 with the same ROI pairs highlighted by black boxes and insignificant correlations indicated by black dots. There were strong correlations between CBF and QTM velocity in the NC group in most regions, including GM, WM, FL, TL, OL, PL, Hippo, and dGM, and moderate correlations between CBF and QTM velocity in the MCI group. In the MCI group, there was a lower correlation between CBF and QTM velocity in almost all evaluated ROIs, demonstrating a mismatch between CBF and QTM velocity in these brain regions with disease progression. The correlations between QTM velocity and CBF in the AD group were generally lower than in the NC group except for PL, but similar or higher than in the MCI group.

### Perfusion linked with cognitive function

**Fig. 6:**
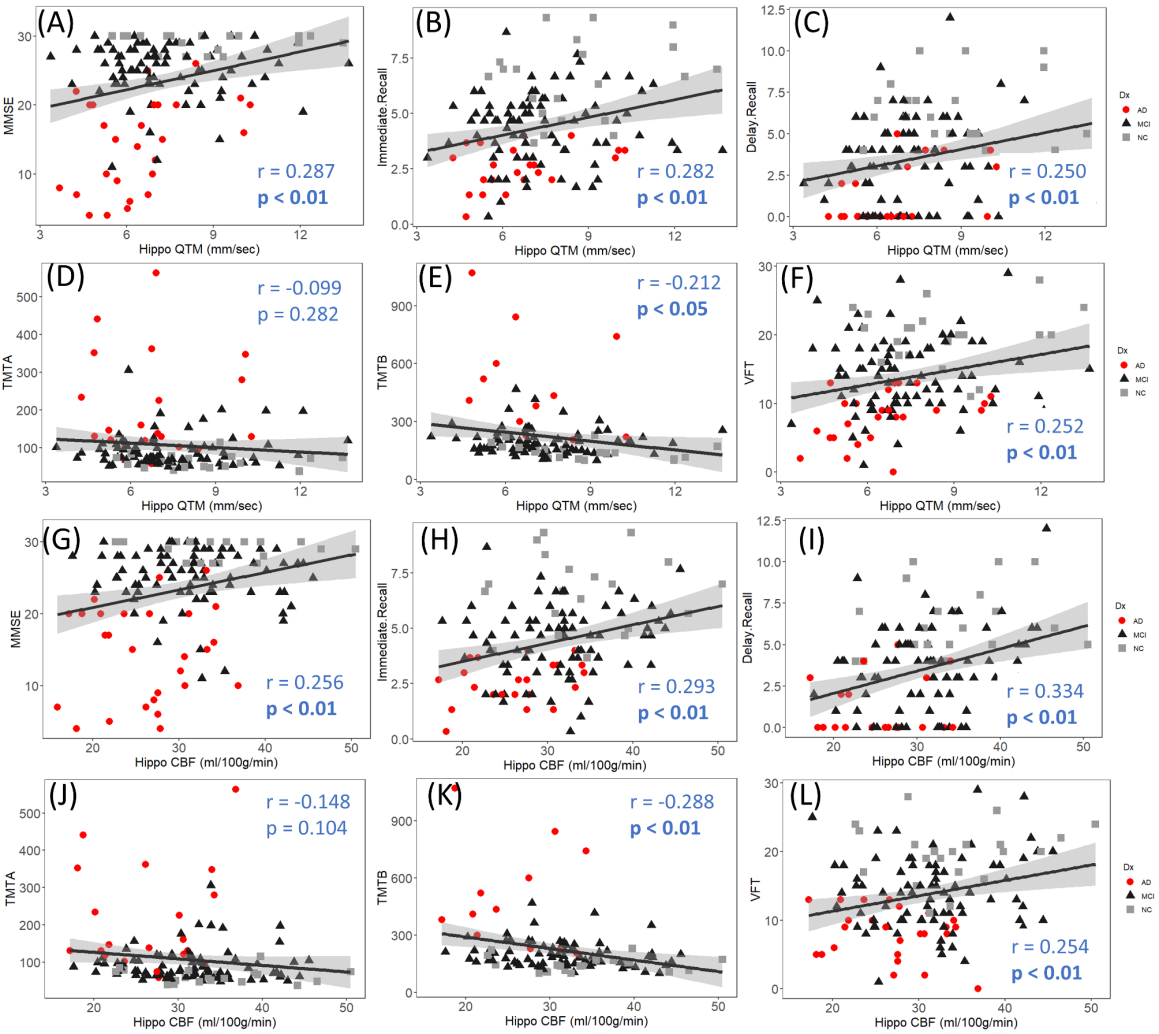
QTM velocity and CBF in the hippocampus with cognitive measures for all subjects. (**A**)-(**F**) are for QTM velocity with MMSE, immediate recall, delay recall, TMT-A, TMT-B, and VFT, respectively; (**G**)-(**L**) are for CBF with same cognitive measurements as for QTM velocity

Correlation analyses were performed to evaluate the relationship between cognitive abilities and perfusion measures in both GM and the hippocampus (see Table 3). Figure 6 (A)-(F) summarize QTM velocity with cognitive measures in the hippocampus, and Fig. 6 (G)-(L) summarize CBF with cognitive measures. QTM velocity in the hippocampus was positively correlated with MMSE (Fig. 6(A) *r* = 0.287, *p* < 0.01), immediate recall (Fig. 6(B) *r* = 0.282, *p* < 0.01), delayed recall (Fig. 6(C) *r* = 0.250, *p* < 0.01), and semantic fluency (Fig. 6(F) *r* = 0.252, *p* < 0.01), and negatively correlated with TMT-B (Fig. 6(E) *r* = -0.212, *p* < 0.01). Similar correlations were observed in GM for QTM velocity with cognitive measures and are summarized in Table 3.

**Table 3:** Correlation of CBF and QTM values with cognition in cortical GM and hippocampus. The top panel is for the whole group and the bottom panel is for non-AD groups

	MMSE		Immediate recall		Delay recall		TMT-A		TMT-B		Semantic fluency		
	r	p	r	p	r	p	r	p	r	p		r	p
**Relationship between CBF (ml/100 g/min) and cognition**													
GM	0.29	< 0.01	0.27	< 0.01	0.28	< 0.01	-0.22	< 0.05	-0.35	< 0.001		0.26	< 0.01
Hippo	0.26	< 0.01	0.29	< 0.01	0.33	< 0.01	*-0.15*	*0.10*	-0.29	< 0.01		0.25	< 0.01
**Relationship between QTM velocity (mm/sec) and cognition**													
GM	0.27	< 0.01	0.26	< 0.01	0.21	< 0.05	*-0.15*	*0.09*	-0.19	< 0.05		0.23	< 0.01
Hippo	0.29	< 0.01	0.28	< 0.01	0.25	< 0.01	*-0.10*	*0.28*	-0.21	< 0.05		0.25	< 0.01
**Relationship between CBF (ml/100 g/min) and cognition excludes the AD group**													
GM	0.02	0.86	0.16	0.12	0.17	0.09	0.01	0.92	-0.24	< 0.05		0.16	0.12
Hippo	0.01	0.97	0.16	0.11	0.25	< 0.05	0.05	0.64	-0.20	0.06		0.18	0.08
**Relationship between QTM velocity (mm/sec) and cognition excludes the AD group**													
GM	0	1	0.17	0.09	0.08	0.42	0.05	0.64	-0.17	0.09		0.11	0.28
Hippo	0.09	0.35	0.21	< 0.05	0.16	0.11	0	0.92	-0.20	< 0.05		0.14	0.16

CBF in the hippocampus was positively correlated with MMSE (*r* = 0.256, *p* < 0.01), immediate recall (*r* = 0.293, *p* < 0.01), delayed recall (*r* = 0.334, *p* < 0.01), and semantic fluency (*r* = 0.254, *p* < 0.01), and negatively correlated with TMT-B (*r* = -0.288, *p* < 0.05), as shown in Fig. 6 (G)-(L). CBF in GM shows similar correlations and is summarized in Table 3. The similar correlation tests were performed for the non-AD group by excluding AD subjects in the analysis and the results were summarized in the bottom panel of Table 3. It showed that most significant correlations in the whole group disappeared in non-AD groups. CBF in GM is correlated with TMT-B (*r* = -0.24, *p* < 0.05) and in the hippocampus is correlated with delay recall (*r* = 0.28, *p* < 0.05). QTM velocity in the hippocampus is correlated with immediate recall (*r* = 0.21, *p* < 0.05) and TMT-B (*r* = -0.20, *p* < 0.05).

## Discussion

Our results demonstrate significant differences in QTM velocity between NC and MCI groups in the GM and hippocampus, while these differences are not observed with CBF, suggesting QTM velocity could constitute an early biomarker for AD. QTM velocity in GM and dGM is sensitive enough to detect perfusion changes regardless of age and sex. Additionally, our results show that both QTM velocity and CBF in the GM and hippocampus are significantly associated with overall cognition (MMSE), immediate recall score, delayed recall score, TMT-B, and VFT. Cerebral perfusion, an indication of blood supply to tissue, is a potential early biomarker of AD and its alteration may appear earlier in AD than other hallmark pathological changes, such as beta-amyloid (Aβ) deposition, hyperphosphorylated tau accumulation, and widespread brain atrophy [41, 42]. 

### CBF and QTM velocity for quantification of blood perfusion

Kety’s CBF and QTM velocity were two measurements for quantification of the blood perfusion in the tissue. CBF measured blood flow (ml/100 g/min), while QTM velocity (mm/sec) measured blood velocity in each voxel. Both measurements can capture the change in blood perfusion. CBF has been widely used to investigate perfusion changes in patients with MCI and AD. Considering patients with developed AD dementia, hypoperfusion was present in most of the brain areas, including GM, OL, FL, PL, TL, amygdala, and hippocampus [43–46]. We observed decreased CBF in GM and hippocampus for patients with AD than NC, which was consistent with previous studies. For comparisons between NC and MCI subjects, a meta-analysis study demonstrated that no obvious changes in CBF in the global, white matter, and GM were identified in MCI subjects [47]. However, some previous studies demonstrated decreased CBF of MCI compared with NC in PL, OL, FL, and TL [45, 46, 48]. Our data show no significant difference in CBF between NC and MCI groups in both GM and dGM. These results demonstrate that CBF is not sensitive enough to detect perfusion change in the early stage of AD.

Previous works have shown that QTM velocity is able to distinguish benign from malignant breast lesions [16] and separate high-grade nonalcoholic fatty liver disease (NAFLD) from low-grade NAFLD using DCE MRI, showing its high sensitivity to subtle physiological change at the early stage of diseases [15]. In this work, QTM velocity calculated from mPLD ASL data is applied to detect cerebral blood perfusion change in AD. Our results found significant differences in QTM velocity between NC and MCI groups in both GM and hippocampus, demonstrating that QTM velocity could be an early AD biomarker.

### Technical issues in the quantification of CBF and QTM velocity

Technically, Kety’s CBF uses a kinetic modeling with a global AIF as an input, and a lump of empirical parameters to evaluate blood perfusion in a voxelwise manner [12, 49]. The problems with CBF include its violation of local mass conservation due to its use of global AIF and its use of only temporal information by ignoring the spatial transport tracer between neighboring voxels [14]. These simplifications of Kety’s model result in the loss of sensitivity of local and spatial changes of the blood perfusion, especially for patients with subtle pathophysiological changes like early-stage AD. Another concern on conventional CBF from multidelay ASL is that the CBF values depend on the number of delays used [50]. Previous studies have shown that multidelay ASL gave lower CBF than single delay ASL [51]. 

In contrast, the QTM model tries to solve both issues in Kety’s model by implementing a biophysical mass transport model with dynamic data. First, QTM doesn’t require an AIF to quantify blood velocity by considering the spatiotemporal derivative of the 4D signal in the formula, which enables us to evaluate both spatial and temporal changes in the dynamic data. Second, QTM doesn’t have assumed parameters that rely on empirical testing, which makes it suitable for all subjects including healthy subjects and patients. The methodological differences between QTM velocity and CBF could be a key reason for better performance of QTM on disease assessment.

Furthermore, both QTM velocity and CBF were used to estimate the perfusion in the tissue. However, the models for calculation of QTM velocity and CBF were different in theory. Therefore, QTM velocity with unite mm/sec and CBF with unit ml/100 g/min measures similar but different physiology of perfusion. As there is autoregulation of cerebral blood flow, the difference between QTM velocity and CBF might be explained by this mechanism [52, 53]. When blood velocity decreases, it can signal a potential reduction in blood flow to the brain. In response, cerebral autoregulation mechanisms can trigger vasodilation (widening of blood vessels) to increase blood flow and restore adequate perfusion [54, 55]. QTM measures the perfusion velocity, which factored out the cross-sectional area from CBF, as CBF is defined as a multiplication of blood velocity and cross-sectional area. At the early stage of disease, the blood flow could be similar to normal but the change of blood velocity and cross-sectional area in capillary might occur within a compensatory/autoregulation mechanism. This could be another reason for the enhanced sensitivity of QTM velocity in distinguishing MCI from NC.

### Perfusion measures associated with age and sex

Our results are consistent with previous studies showing that CBF decreases with age in GM but not in dGM [56]. QTM velocity decreases with age in both the GM and dGM, demonstrating a higher sensitivity than CBF for detecting perfusion changes. CBF values in the dGM are higher in females than males and in GM are relatively similar. A previous study shows that females exhibit significantly higher CBF values when compared to males [57]. These contradicting results may be due to sample variation. The previous study includes subjects aged 20 to 80, while our study includes older subjects aged 55 to 90 [57]. These results indicate that perfusion is linked to age and sex, and that QTM velocity is more sensitive than CBF in the detection of age- and sex-related alteration in perfusion. To eliminate these effects when comparing diagnostic groups, age and sex served as covariates.

### Correlations between QTM velocity and CBF

We observed strong correlations (*r* > 0.55) between QTM velocity and CBF in the NC group across many evaluated ROIs. In the MCI group, this association decreased to moderate (r ~ = 0.45) in the same ROIs. As the disease progresses, the correlations between QTM velocity and CBF change across NC, MCI, and AD. The potential reasons for this correlation change may be attributed to two main factors. First, the global AIF in CBF might be sufficient in NC subjects since their blood perfusion is high and the local estimated CBF is not affected much due to systematic estimation error. However, in MCI subjects with pathophysiological change in vasculature and perfusion, the disadvantage of global AIF starts to play a role and reduces the sensitivity and accuracy of CBF, due to alterations in blood perfusion pathway or quantity. On the other hand, QTM does not rely on AIF to estimate the blood perfusion and thus offers high sensitivity in estimating blood velocity and its subtle change. Second, the decreased perfusion in MCI patients compared with NC could be due to spatial changes in vasculature structure and blood perfusion routes. CBF is fitted from the ASL data using Kety’s model in a voxelwise manner that only considers the temporal relationship between data frames, while the QTM model based on biophysical principles, utilizes spatiotemporal information of dynamic data to explore the transport of tracer in blood across voxels. QTM model utilizes the same data as Kety’s model more efficiently, and may be the second reason why QTM is the more sensitive measure. From the correlation maps, we also observe that some brain regions are more affected than others during the disease development in AD, which needs further investigation of the blood route supply alteration in the AD spectrum.

### Decreased CBF and QTM velocity linked with cognition

The hippocampus is considered a major player in memory. Hippocampal atrophy is an established imaging biomarker in AD and constitutes neurodegeneration in the A/T/N framework [58, 59]. However, blood perfusion decline occurs much earlier than the appearance of brain atrophy [60]. In the hippocampus, we found decreased CBF and QTM velocity in the AD group compared to the MCI and the NC groups. Moreover, QTM velocity showed a significant difference between the MCI and NC groups, demonstrating hippocampal perfusion changes at the early stage of AD, and was not seen using conventional CBF. Our results also showed that CBF and QTM velocity in the hippocampus correlated with cognition, including global cognition, memory, executive function, and language. After excluding the AD group in the analysis, most significant correlations disappeared but between QTM velocity in the hippocampus and immediate recall and TMT-B were still significant. These findings confirmed that blood perfusion coupled with blood flow velocity in the hippocampus can be useful diagnostic markers of AD.

Glymphatic function has been proposed to explain the brain clearance deficits in AD. Blood perfusion may play a key role in the general glymphatic flow, whose deficits have been shown to be the cause of Aβ accumulation. [61–64] QTM velocity from the labeled freely diffusible water signal in ASL may reflect the total fluid transport in the brain, including blood flow in vascular space and fluid flow in perivascular and interstitial spaces [65]. The content of glymphatic flow information in QTM velocity derived from ASL would require further investigation.

### Limitations of this study

We recognize several limitations in this study. First, the relatively small sample size in NC and AD groups. A large-scale prospective study, containing participants from subjective cognitive decline is needed to further explore the underlying mechanisms of blood flow velocity changes. Second, the ground truth of blood flow and blood flow velocity of the brain is unknown, although we had performed numerical simulations in the kidney and liver to confirm the accuracy of QTM. Due to the complexity of brain microvasculature, it is challenging to conduct numerical simulations of QTM in the brain as what has been done in the kidney and liver. Alternatively, we are performing a deep learning-based microvasculature and QTM quantification study (QTMnet), which could help to overcome the difficulty of brain blood flow simulation [66]. Third, PET imaging data is not available, thus the association between QTM velocity, brain clearance, and Aβ deposition is not yet investigated. Finally, the nature of cross-sectional study limits us to study different subjects at different disease stages. Our near goal is to run a longitudinal study using QTM velocity to further study its underlying mechanism for early detection in AD. Future research will include PET data and explore the association between QTM velocity and brain Aβ deposition to design longitudinal studies.

## Conclusions

This study demonstrated a reduced QTM velocity in GM and the hippocampus in MCI patients compared with NC, suggesting QTM velocity is a potential early biomarker for AD. Decreased CBF and QTM velocity in the hippocampus correlated with cognitive decline. These findings contribute to an improved understanding of perfusion change and cognitive decline in AD.

### Electronic supplementary material

Below is the link to the electronic supplementary material.

Supplementary Material 1

## Data Availability

The data that support the findings of this study are available on request from the corresponding author when the appropriate data sharing agreements are consented.

## Abbreviations

AD: Alzheimer’s disease
CBF: Cerebral blood flow
MRI: Magnetic resonance imaging
DCE: Dynamic contrast enhanced
DSC: Dynamic susceptibility contrast
ASL: Arterial spin labeling
GBCA: Gadolinium-based contrast agent
AIF: Arterial input function
QTM: Quantitative transport mapping
MCI: Mild cognitive impairment
NC: Normal control
MMSE: Mini-mental state examination
CDR: Clinical dementia rating
TMT: Trail Making Test
RAVLT: Rey Auditory Verbal Learning Test
VFT: Verbal fluency test
PLD: Post labeling delay
T1W: T1 weighted image
T2W: T2 weighted image
FS: FreeSurfer
GM: Cerebral cortex
WM: Cerebral white matter
dGM: Deep gray matter
TL: Temporal lobe
FL: Frontal lobe
PL: Parietal lobe
OL: Occipital
Hippo: Hippocampus
PVE: Partial volume effect
MNI: Montreal Neurological Institute
PCASL: Pseudo-continuous ASL
