# Supplementary material for: Quantitative transport mapping of multi-delay arterial spin labeling MRI detects early blood perfusion alterations in Alzheimer’s disease
Source: Alzheimers Res Ther. 2024 Jul 8;16:156. doi: 10.1186/s13195-024-01524-6 (PMC11229285; doi:10.1186/s13195-024-01524-6)
Supplement: Supplementary file 1 — Supplementary Material 1 [file 13195_2024_1524_MOESM1_ESM.docx]

# Supplementary material

## Kinetic model of multiple delay ASL for CBF quantification

The general kinetic model of arterial spin labeling (ASL) for CBF quantification is derived in the well-known Buxton’s paper. [1] For multiple delay ASL, the derived formula for CBF estimation is as follows: [2, 3]

$$\Delta M=\left\{ \begin{matrix} 0 & 0<LD+PLD<ATT \\ \frac{2\cdot\alpha\cdot\alpha_{BS}\cdot T_{1b}\cdot M_{0a}\cdot CBF\cdot e^{-\frac{ATT}{T_{1b}}}\cdot(1-e^{-\frac{LD+PLD-ATT}{T_{1b}}})}{6000} & ATT<LD+PLD<ATT+LD \\ \frac{2\cdot\alpha\cdot\alpha_{BS}\cdot T_{1b}\cdot M_{0a}\cdot CBF\cdot e^{-\frac{PLD}{T_{1b}}}\cdot(1-e^{-\frac{LD}{T_{1b}}})}{6000} & ATT<PLD \end{matrix} \right.$$

where $\Delta M$ is the signal difference between the pair of control and label images, LD the labeling duration, PLD the post labeling delay time, ATT the arterial transition time, $\alpha$ the labeling efficiency, $T_{1b}$ the longitudinal relaxation time of the arterial blood, $M_{0a}$ the equilibrium magnetization of arterial blood calculated as $M_{0a}=\frac{S_{PD}}{\lambda}$ with $S_{PD}$ the proton density weighted image and $\lambda$ the tissue-blood partition coefficient, the factor 6000 converts the units for CBF from ml/g/s to ml/100g/min.

To estimate CBF from the above kinetic model with multiple PLDs, a Bayesian inference framework was used by implementing all the data from multiple PLDs in the model, in which an iterative algorithm that minimizes a free energy term based on an approximation to the true posterior distribution for the model parameters given the data and kinetic model chosen. [4] The implementation of these approaches was built in the BASIL package in the FSL toolbox. [5]

## Region of interest for perfusion measurements

We have overlayed the region of interests on the T1W brain to show their segmentation and location as shown in Figure S1. Specifically, the ROIs used in this study are defined by FreeSurfer (FS) look-up-table (LUT) as shown in Table S1.


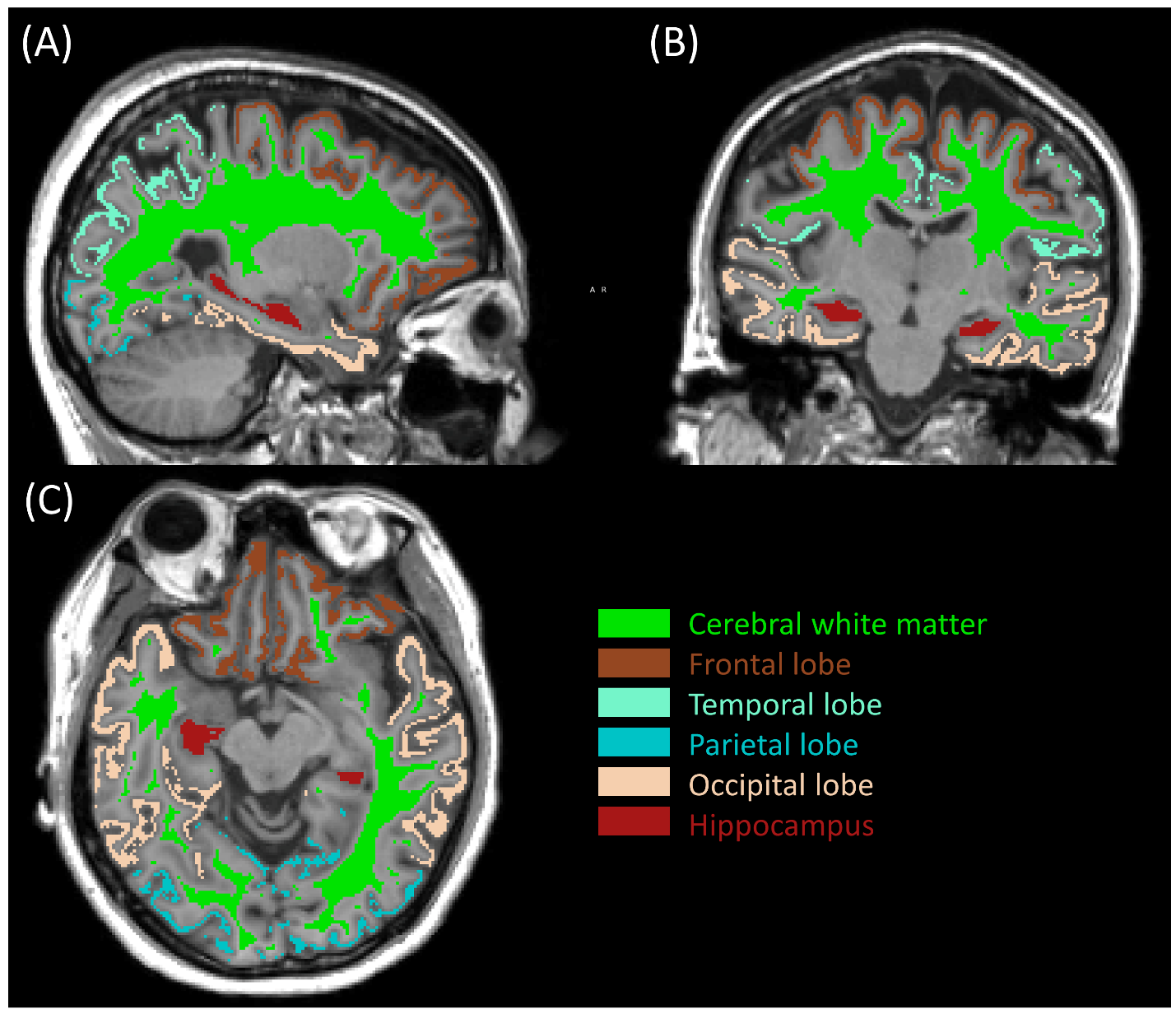


**Figure S1. The investigated ROIs overlayed on T1w.** (A) Sagittal view; (B) Coronal view; (C) Axial view. Gray matter in the main paper refers to the cerebral cortex region, which is a combination of four cortical lobes including the frontal lobe, temporal lobe, parietal lobe, and occipital lobe.

**Table S1. The definition of ROIs used in this study using FreeSurfer look-up-table.** LUT is at : <https://surfer.nmr.mgh.harvard.edu/fswiki/FsTutorial/AnatomicalROI/FreeSurferColorLUT>.

| ROI name | FS LUT label |
| --- | --- |
| Cerebral Cortex (GM) | 1001-1035, 2001-2035 |
| Cerebral White Matter (WM) | 2, 41 |
| Frontal Lobe (FL) | 1002, 1003, 1012, 1014, 1017, 1018, 1019, 1020, 1024, 1026, 1027, 1028, 1032, 2002, 2003, 2012, 2014, 2017, 2018, 2019, 2020, 2024, 2026, 2027, 2028, 2032 |
| Temporal Lobe (TL) | 1001, 1006, 1007, 1009, 1015, 1016, 1030, 1033, 1034, 2001, 2006, 2007, 2009, 2015, 2016, 2030, 2033, 2034 |
| Parietal Lobe (PL) | 1008, 1010, 1022, 1023, 1025, 1029, 1031, 2008, 2010, 2022, 2023, 2025, 2029, 2031 |
| Occipital Lobe (OL) | 1011, 1013, 1005, 1021, 2011, 2013, 2005, 2021 |
| Deep Gray Matter (dGM) | 10, 11, 12, 13, 17, 18, 26, 49, 50, 51, 52, 53, 54, 58 |
| Hippocampus (Hippo) | 17, 53 |

## Comparison between CBF and QTM velocity in subject space

To compare the pattern difference between CBF and QTM velocity, we have shown the QTM velocity and CBF map for three example subjects (NC, MCI, and AD) as shown in Figure S2. Note that all of the three subjects are male and 70 years old. We can observe that both QTM velocity and CBF map decrease from NC to MCI and AD. More specifically, the CBF map is smoother than the QTM velocity in the same subject. The smooth CBF maps caused by smoothing filter and using smoothed M0 image in CBF reconstruction might have more partial volume effect. QTM that benefits from the use of L1 regularization and dynamic data in the method gives sharp velocity map. These differences between the two methods might help to understand their efficacy on patients’ diagnosis.


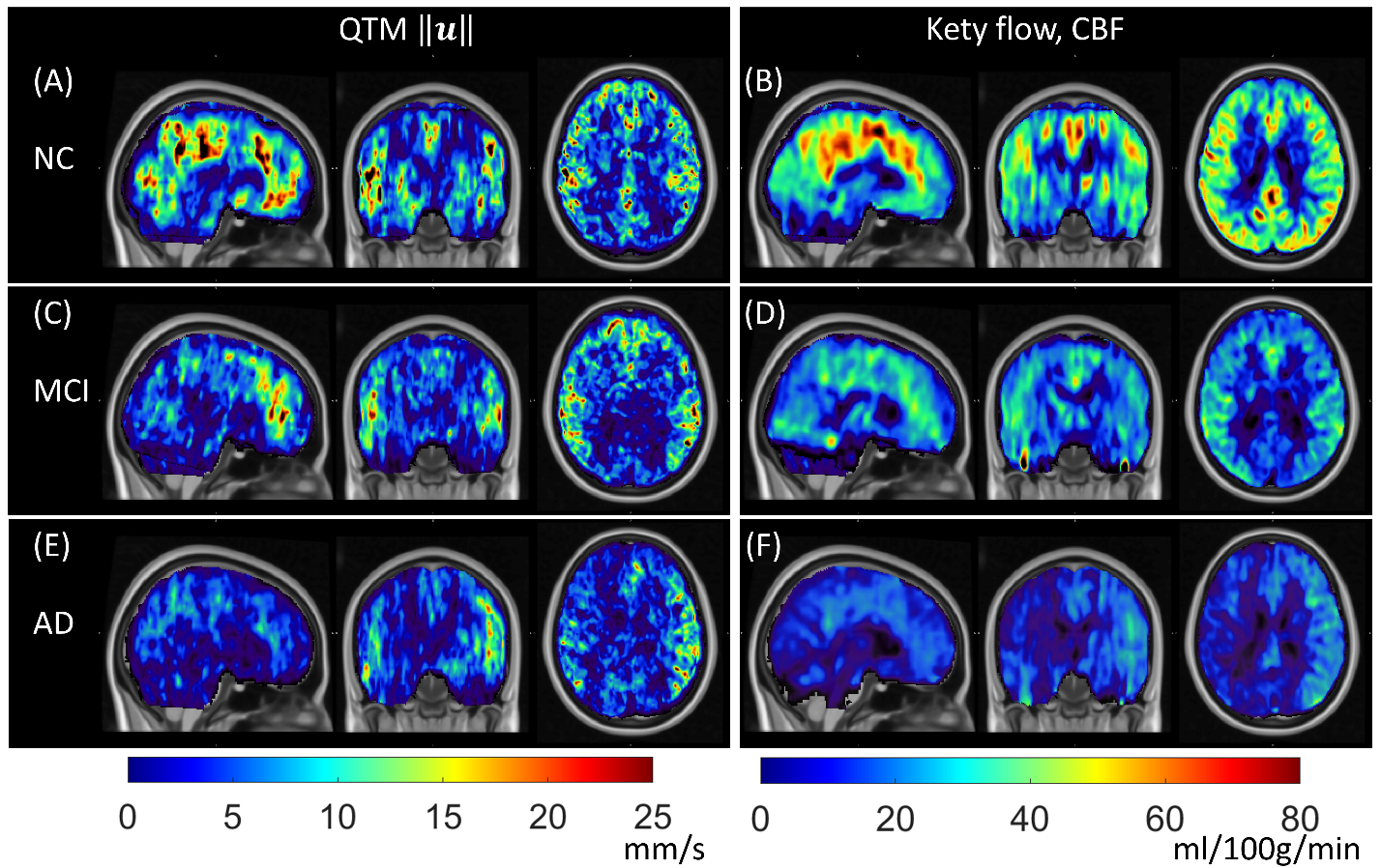


**Figure S2. The example of QTM velocity and CBF map at subject level with 3 subjects (NC, MCI, and AD) all male and 70 years old.** (A) and (B) are QTM velocity and CBF map for a NC subject; (C) and (D) are QTM velocity and CBF map for a MCI subject; (E) and (F) are QTM velocity and CBF map for an AD subject. Note that all QTM maps are at the same value range [0, 25] mm/s, and all CBF maps are at the same value range [0, 80] ml/100g/min.

## References

1. Buxton, R.B., et al., *A general kinetic model for quantitative perfusion imaging with arterial spin labeling.* Magn Reson Med, 1998. **40**(3): p. 383-96.

2. Woods, J.G., et al., *Recommendations for quantitative cerebral perfusion MRI using multi-timepoint arterial spin labeling: Acquisition, quantification, and clinical applications.* Magn Reson Med, 2024.

3. Chappell, M.A., et al., *Separation of macrovascular signal in multi-inversion time arterial spin labelling MRI.* Magn Reson Med, 2010. **63**(5): p. 1357-65.

4. Chappell, M.A., et al., *Variational Bayesian Inference for a Nonlinear Forward Model.* Ieee Transactions on Signal Processing, 2009. **57**(1): p. 223-236.

5. Chappell, M.A., et al., *BASIL: A toolbox for perfusion quantification using arterial spin labelling.* Imaging Neuroscience, 2023. **1**: p. 1-16.
